# Laparoscopic pyeloplasty and isthmusectomy in horseshoe kidney with ureteropelvic junction obstruction: a case report and review of the literature

**DOI:** 10.1186/s13256-026-06020-z

**Published:** 2026-05-27

**Authors:** Tuan Thanh Nguyen, Minh Sam Thai, Quy Thuan Chau, Khac Chuan Hoang, Kinh Luan Thai, Le Quy Van Dinh, Quoc Bao Nguyen, Anh Ly, Dang Nhat Minh Pham, Hoai Tam Ly, Xuan Thai Ngo

**Affiliations:** 1 University of Medicine and Pharmacy at Ho Chi Minh City, 217 Hong Bang Street, Ward 11, District 5, Ho Chi Minh City, Vietnam; 2 Department of Urology, Cho Ray Hospital, Ho Chi Minh City, Vietnam; 3 Department of Urology, University of California Irvine, Orange, USA; 4 Pham Ngoc Thach University of Medicine, Ho Chi Minh City, Vietnam

**Keywords:** Horseshoe kidney, Ureteropelvic junction obstruction, Laparoscopic pyeloplasty, Hynes–Anderson, Isthumusectomy, Endoscopic gastrointestinal anastomosis (GIA) stapler

## Abstract

**Background:**

Ureteropelvic junction (UPJ) obstruction is commonly observed in patients with horseshoe kidneys due to congenital anatomical anomalies, presenting unique challenges in both diagnosis and surgical management. The role of isthmusectomy in laparoscopic reconstructive surgery for this condition remains a topic of ongoing debate. This report outlines our initial experience with a minimally invasive approach to managing UPJ obstruction in a horseshoe kidney patient, utilizing Anderson–Hynes dismembered pyeloplasty combined with isthmusectomy.

**Case presentation:**

A 28-year-old Vietnamese male was admitted with recurrent right flank pain and fever. A known diagnosis of congenital horseshoe kidney was noted. Contrast-enhanced computed tomography revealed a horseshoe kidney with grade III right-sided hydronephrosis and an atrophic left kidney. Diuretic renal scintigraphy with DTPA revealed a decline in glomerular filtration rate (GFR) in both kidneys, with values of 7.97 mL/min (15.8%) on the left side and 42.5 mL/min (84.2%) on the right side, accompanied by significant right-sided obstruction evidenced by a delayed half-time clearance exceeding 30 min.

A transperitoneal laparoscopic approach was selected for surgical reconstruction. Intraoperatively, a lower pole vessel was found crossing anterior to the right ureteropelvic junction (UPJ), causing compression and obstruction. In addition, the renal isthmus was located posterior to the ureter, resulting in kinking. Isthmusectomy was performed using an endoscopic stapler to relieve the anatomical distortion. The right ureter was transected, relocated anterior to the crossing vessel, and reconstructed using the Anderson–Hynes dismembered pyeloplasty technique over a pre-inserted double-J stent.

The procedure lasted 270 min with an estimated blood loss of 50 mL. The drain was removed on postoperative day 3, and the patient was discharged on day 5 without complications. At 3-month follow-up, the patient remained asymptomatic. Postoperative imaging demonstrated preserved renal function and significant improvement in right-sided urinary drainage, with reduced hydronephrosis and improved excretory function on follow-up DTPA renal scintigraphy.

**Conclusions:**

Transperitoneal laparoscopic Anderson–Hynes pyeloplasty combined with isthmusectomy appears to be a feasible and safe option for managing ureteropelvic junction obstruction in patients with horseshoe kidney, particularly in cases involving vascular compression or isthmus-related obstruction, when performed by experienced surgeons.

## Introduction

Horseshoe kidney is the most common congenital fusion anomaly, with the prevalence being 1/400 of births [1]. They are more common in males, reported ratio of 2:1 to females [2, 3]. Although the majority of patients with horseshoe kidneys are asymptomatic, ureteropelvic junction obstruction (UPJO) occurs in as many as one-third of individuals with horseshoe kidneys [1, 4–6]. The following factors may contribute to UPJO in horseshoe kidney: abnormal blood vessel supply to the kidney, high insertion of the ureter, its abnormal course across the renal isthmus, and its intrinsic pathogenesis [1].

While endoscopic (retrograde or antegrade) management of UPJO remains the least invasive option, it is often associated with lower success rates. Laparoscopic pyeloplasty has emerged as a safe and effective reconstructive technique for managing UPJO in patients with horseshoe kidney. Compared with open surgery, it offers comparable clinical outcomes, improved postoperative pain control, shorter recovery time, and a reduced risk of hemorrhagic complications when performed by experienced surgeons [7, 8]. The role of isthmusectomy remains debated, this procedure may improve ureteropelvic junction patency by repositioning the kidneys and reducing medial compression [9]. However, it carries increased risks, including bleeding and renal infarction, especially in patients with prior surgery or recurrent hydronephrosis. Consequently, many surgeons advocate for a conservative approach, as comparable surgical outcomes are often achievable without isthmusectomy [10].

In this context, we report a case of ureteropelvic junction obstruction in a patient with a horseshoe kidney, highlighting our initial experience with Anderson–Hynes dismembered pyeloplasty combined with isthmusectomy and its early postoperative outcomes.

## Case presentation

A 28-year-old Vietnamese male presented with a 3-month history of recurrent right flank pain and a documented fever of 38.5 °C. Vital signs were otherwise within normal limits upon admission. The patient had no significant past medical history, with no previous surgical interventions and no known familial history of urological or renal disorders. Physical examination revealed right costovertebral angle tenderness. Contrast-enhanced computed tomography (CT) demonstrated a horseshoe kidney with bilateral grade III hydronephrosis (according to The Society for Fetal Urology) and atrophy of the left kidney.

The initial diagnosis included bilateral infectious hydronephrosis due to ureteropelvic junction obstruction (UPJO) in the setting of a horseshoe kidney, associated with acute kidney injury (serum creatinine: 1.76 mg/dL). The patient was treated with broad-spectrum antibiotics and bilateral retrograde double-J stent placement for decompression.

**Fig. 1:**
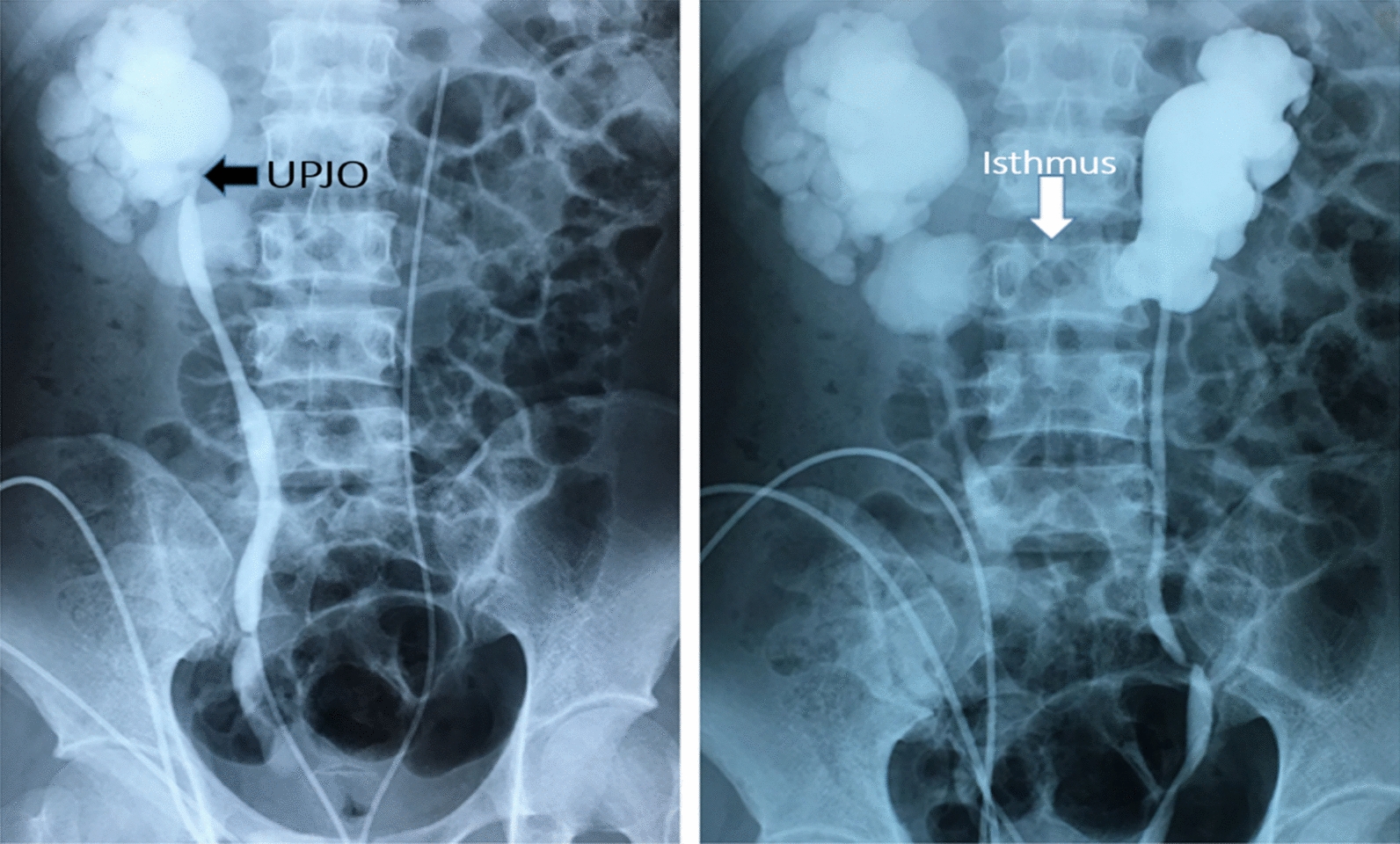
Retrograde pyelography; Left panel: black arrows denotes right UPJO position. Right panel: white arrow denotes the isthmus of horseshoe kidney

Two months after stabilization, the patient showed no clinical or laboratory signs of infection. Urinalysis and white blood cell count were within normal limits, urine cultures were negative, and baseline serum creatinine was 1.5 mg/dL. Diuretic renal scintigraphy using DTPA demonstrated a glomerular filtration rate (GFR) of 42.5 mL/min (84.2%) for the right kidney and 7.97 mL/min (15.8%) for the left kidney. A severe obstruction of the right urinary tract was confirmed by Furosemide washout test, with a half-life clearance time exceeding 30 min. Retrograde pyelography revealed a horseshoe kidney with the isthmus located at the L3 vertebral level and right-sided UPJ obstruction (Fig. 1). Based on these findings, a transperitoneal laparoscopic approach was selected for reconstructive surgery.

**Fig. 2:**
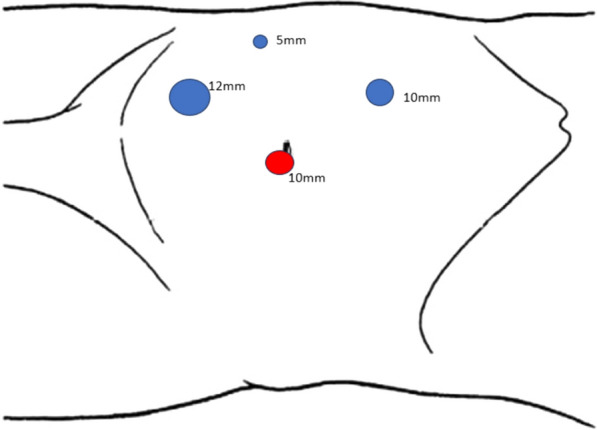
Port placement. The red color was camera trocar (umbilical trocar)

The patient was positioned in a slight oblique supine position with moderate flexion of the operating table at the level of the contralateral kidney. The upper arm was adducted alongside the torso. A four-port transperitoneal laparoscopic approach was utilized (Fig. 2).

The procedure began with mobilization of the hepatic flexure and dissection of Toldt’s fascia to expose the right ureter. Intraoperatively, the right ureter was found to cross anteriorly over the isthmus of the horseshoe kidney. A lower pole renal vessel was observed compressing the right ureteropelvic junction (UPJ), consistent with the cause of right-sided hydronephrosis. The isthmus was subsequently exposed and transected using a 60 mm Endoscopic GIA linear stapler. Dissection of the ureter and renal isthmus was technically challenging due to dense adhesions involving surrounding structures, attributed to prior infection and interventions. These difficulties significantly prolonged the operative time.

**Fig. 3:**
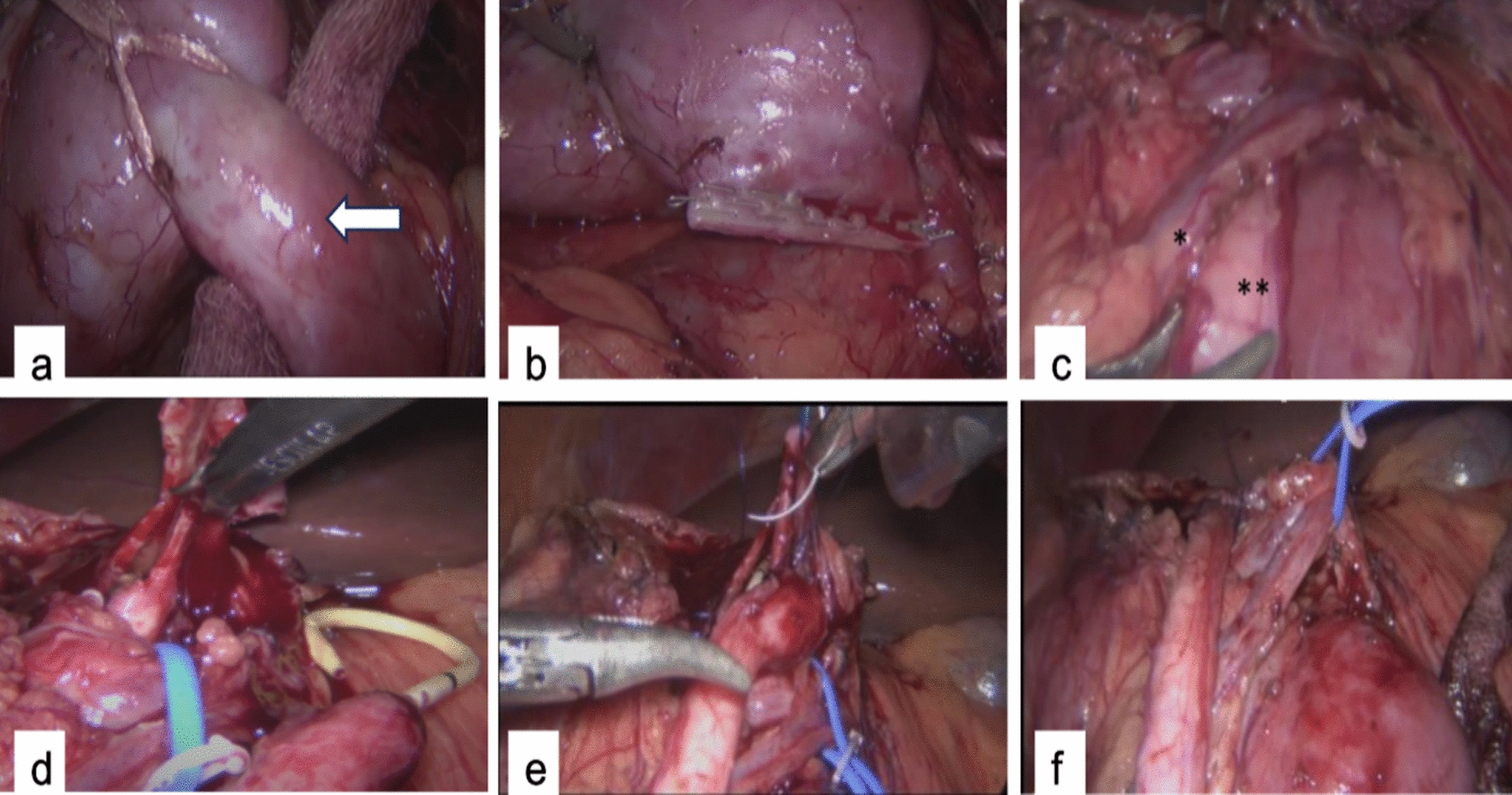
Step-by-step surgical procedure. **a** Isthmus of the horseshoe kidney was exposed (white arrow). **b** Isthmusectomy was performed using an 60 mm endoscopic GIA linear stapler. **c** Lower pole renal vessel (*) was seen crossing anterior to and compressing the ureteropelvic junction (**). **d** Ureter was transected, and the pathological segment was excised; a pre-inserted double-J stent was maintained in position during the procedure. **e** Ureteropelvic anastomosis was performed using PDS 4–0 sutures. **f** Reconstructed ureteropelvic junction was relocated anterior to the crossing lower pole vessel

The right ureter was then transected and transposed anteriorly to the crossing vessel. A preoperatively placed double-J stent was retained, and ureteropelvic reconstruction was performed using the Anderson–Hynes dismembered pyeloplasty technique. The anastomosis was completed with PDS 4–0 sutures with a double-J stent inside. A peritoneal drain was placed adjacent to the ureteral repair (Fig. 3).

The total operative time was 270 min, with an estimated blood loss of 50 mL. The drain was removed on postoperative day 3, and the patient was discharged on day 5 in stable condition. At 1-month follow-up, bilateral double-J stents were removed. The patient remained asymptomatic, and at 3 months postoperatively, he reported no clinical complaints.

**Fig. 4:**
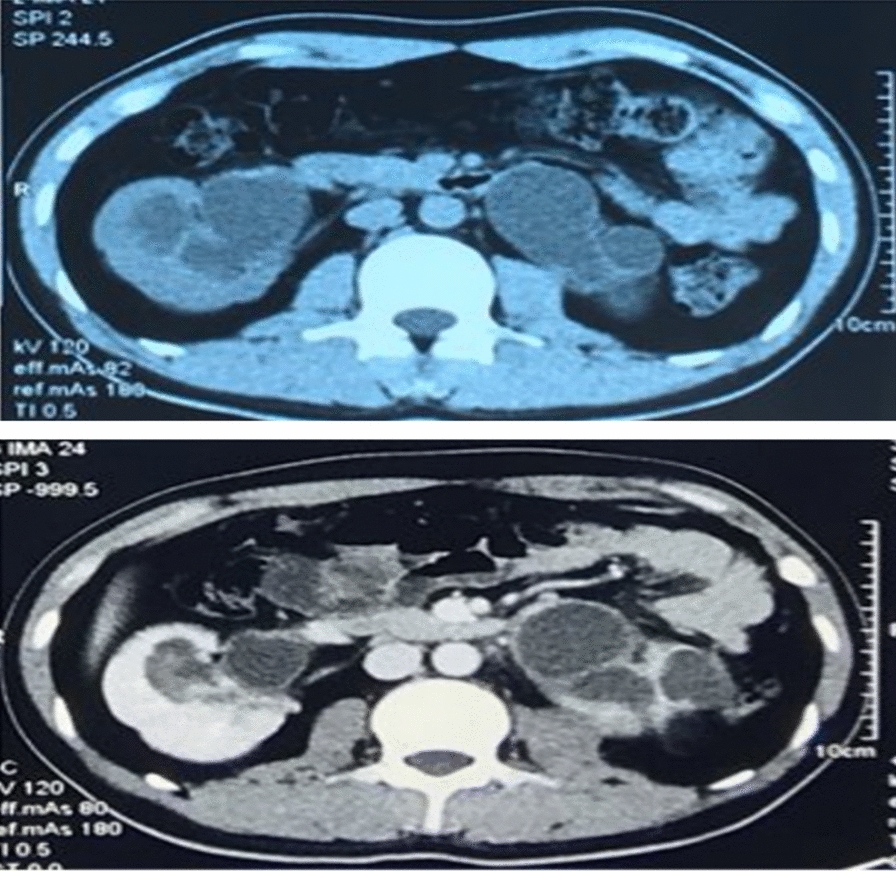
Preoperative contrast-enhanced CT scan of the horseshoe kidney and postoperative follow-up image after 3 months. Horseshoe kidney with grade III right hydronephrosis and atrophy left kidney (above), and the diminished hydronephrosis (grade II) after 3 months (below)

**Fig. 5:**
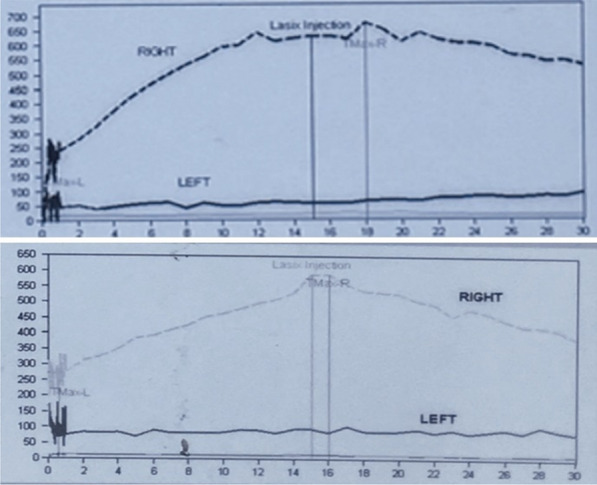
Timeline summarizing clinical progression, diagnostic modalities, therapeutic interventions, and patient follow-up

**Fig. 6:**
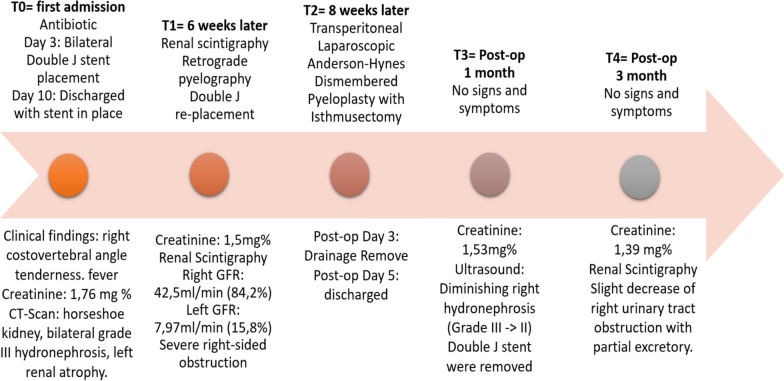
Preoperative diuretic renal scintigraphy with DTPA of the horseshoe kidney showed the servered obstruction of the right kidnet with half-life clearance time more than 20 min and markedly decreased left kidney function (above) and postoperative follow-up after 3 months figured out the obstruction of the right urinary system after surgery was improved with the excretory phase curves demonstrated prompt drainage

Follow-up contrast-enhanced CT scan demonstrated a remarkable reduction of right-sided hydronephrosis, decreasing from Grade III to Grade II (Fig. 4). At 3 months postoperatively, serum creatinine levels experienced a downward trend to 1.39 mg% compared to 1.53 mg% pre-op. DTPA renal scintigraphy showed preserved overall renal function compared to preoperative values (Fig. 5). Notably, drainage of the right renal unit improved significantly, with a marked reduction in excretion delay, although the half-time clearance remained slightly prolonged (> 20 min) (Fig. 6).

## Discussion

Horseshoe kidney is one of the most common fusion abnormality of renal embryogenesis development, which is found in approximately 0.25% of the general population [1]. The condition is defined by the presence of two anatomically distinct renal units located on either side of the vertebral column, fused at their lower poles by a parenchymal or fibrous isthmus that traverses the midline of the body [11]. The renal pelvis and ureters in horseshoe kidney are more anterior than normal, with the ureters passing anterior to the isthmus. This altered course predisposes the ureters to kinking or angulation, which may lead to hydronephrosis [12]. Most horseshoe kidney cases are asymptomatic and do not require intervention unless complications, such as infection, urolithiasis, or UPJO. Hydronephrosis is a common radiographic finding with horseshoe kidneys, this can be secondary to non-refluxing and non-obstructing etiology. Hydronephrosis in horseshoe kidney due to UPJO occurs in about 15–33% of cases [1, 4–6, 13]. UPJO is considered to develop secondary to congenital stenosis, high ureteral insertion, abnormal ureteral passage through the isthmus, compressing vessels supplying the isthmus, or abnormal mobility of the UPJ segment [1]. The risk of formation urolithiasis, which occurs in 21–60% of patients [5, 14], as well as linking these anatomical abnormalities, which occur in up to 30% of these patients were also postulated to be a reason for this condition [15].

Several etiological factors have been implicated in the development of ureteropelvic junction obstruction (UPJO), particularly in patients with horseshoe kidney. As a result, a variety of surgical and minimally invasive treatment approaches have been proposed, including conventional dismembered pyeloplasty, Scardino or Foley Y-V pyeloplasty with or without isthmusectomy and nephropexy, vascular hitch (Hellstrom’s technique), percutaneous and retrograde endopyelotomy [16–19].

The standard surgical approach for managing ureteropelvic junction obstruction (UPJO) in patients with a horseshoe kidney traditionally involves open dismembered pyeloplasty using the Anderson–Hynes technique, frequently accompanied by isthmusectomy and nephropexy of the affected renal unit [20]. Minimally invasive surgical techniques have transformed the management of various urological disorders, including ureteropelvic junction obstruction (UPJO) in patients with horseshoe kidney (HSK). In recent years, both laparoscopic and robot-assisted pyeloplasty have emerged as effective alternatives to open surgery, demonstrating favorable clinical outcomes in appropriately selected cases. These approaches are associated with comparable success rates, improved postoperative pain control, shorter hospital stays, accelerated recovery, and a lower incidence of perioperative complications—particularly when performed by experienced surgeons [21, 22].

Despite the clinical benefits of robotic surgery, its widespread adoption remains limited in resource-constrained settings due to substantial financial burdens. The high cost of robotic platforms and limited insurance coverage in many developing countries hinder access to this technology. Consequently, conventional laparoscopic pyeloplasty is widely regarded as a cost-effective and reliable option for managing UPJO in patients with HSK. However, while early results are encouraging, the existing literature includes a relatively small number of reported cases, highlighting the need for further studies to confirm long-term safety and efficacy [21, 22].

Laparoscopic pyeloplasty can be performed using either a transperitoneal or retroperitoneal approach, both of which have demonstrated high success rates and low complication rates [23]. In cases of horseshoe kidney, where the renal pelvis and ureteropelvic junction (UPJ) are typically located anterior to the isthmus, the transperitoneal approach is generally preferred due to easier anatomical access [24]. This approach also provides a spacious operative field, allowing for clear dissection of the ureter and associated vessels, which facilitates the transposition of these structures when indicated. In contrast, retroperitoneal laparoscopic pyeloplasty has been less commonly utilized in the management of UPJO in horseshoe kidneys [22]. While this technique offers the advantage of avoiding intra-abdominal adhesions, it presents technical challenges. Specifically, maintaining an adequate working space can be difficult due to the limited retroperitoneal volume and the challenge of confining carbon dioxide insufflation within the retroperitoneal cavity [25].

Crossing renal vessels are accounting for at least 40% of adults with primary UPJO [1]. The old concept was originally recommended by Hellstrom, by repositioning crossing vessels cephalad and building upon the less invasive surgical exposure [17]. Where applicable, vascular relocation without pyeloplasty carries some potential advantages, including short operative time, shortened hospital stay, and avoidance of reconstruction complications, such as stricture, urine leak, and stent removal [16]. Anderson–Hynes technique, which can remove of the intrinsic obstructive segment and anastomosis ureter in front of the compressing vessels or isthmus, while the Foley Y-V technique is preferable for high insertion of the ureter and tiny pelvis [16].

Endopyelotomy was introduced for UPJO management in horseshoe kidney with a success rate ranging from 66% to 75% based on three small series [26–28], which range from 77% to 89% without crossing vessels cause, and less than 70% in the presence of a crossing vessel. The presence of crossing vessels is a predictor of the failure rate [29].

The role of isthmusectomy in the surgical management of ureteropelvic junction obstruction (UPJO) in patients with a horseshoe kidney remains a subject of ongoing debate. Culp et al. suggested that isthmusectomy may facilitate improved drainage by allowing the renal units to assume a more dependent position postoperatively. This anatomical shift may enable the medial portion of the kidney to rotate, thereby reducing the likelihood of recurrent obstruction [14]. The decision to perform an isthmusectomy in patients with a horseshoe kidney is guided by a range of clinical considerations. Current literature identifies several primary indications for this procedure, including obstructive uropathy and urolithiasis, symptomatic vascular compression, and the presence of benign or malignant lesions within the isthmus. Additional indications may include cosmetic concerns or the need to facilitate ancillary procedures, such as pyeloplasty, particularly when the isthmus contributes to anatomical distortion or impedes surgical access [21].

However, multiple studies have demonstrated favorable surgical outcomes without the need for isthmus division, raising questions about its routine use [16, 21, 30, 31]. Furthermore, concerns persist regarding the potential risks associated with isthmusectomy, including significant intraoperative bleeding and renal infarction due to the presence of aberrant vascular supply to the isthmus [10]. Given these risks, isthmusectomy may not be the optimal surgical approach in all cases and should be considered selectively based on individual anatomical and pathology [10].

In our case, the patient experienced persistent symptoms, such as urinary tract infection, costovetebral pain, and renal impairment. Intraoperative assessment revealed that the right ureter was significantly angulated and kinked due to extrinsic compression by the renal isthmus, resulting in proximal ureteral dilatation. Given the presence of this mechanical obstruction, isthmusectomy was performed to relieve ureteral compression, enable proper anatomical realignment, and restore effective urinary drainage while preserving renal function. The procedure was performed using a 60 mm endoscopic gastrointestinal anastomosis (GIA) stapler, which has been reported as a safe and effective tool for renal isthmus transection [32]. This device enables rapid and controlled division with concurrent vascular sealing, thereby minimizing blood loss and reducing operative time. Its application is particularly beneficial in minimally invasive procedures, where precise and hemostatic tissue handling is critical to optimizing surgical outcomes [32, 33].

Because of limited data on the safety, and efficacy of laparoscopic pyeloplasty with isthmusectomy in the management of UPJO in horseshoe kidney, we also summarize the literature that is based on the evidence for this approach. Feddegon, et al. reported that 9 cases of reconstructive pyeloplasty for UPJO in horseshoe kidney with clinical and radiographic success rates of 100% and 78%, respectively. [34] The mean operative time was 136 min and the maximum amount of blood loss was 50 ml and no major complications were recorded. In addition, Bove et al. showed that in their study with Anderson–Hynes technique, the success rate was 100% and the average op-time was 196 min with no complications [35]. In 2014, Ping Wang et al. presented a retroperitoneal laparoscopic approach for pyeloplasty and isthmusectomy to treat UPJO in horseshoe kidney with clinical and radiographic rate accounting for 100% with no complications and minimal blood loss was 20–40 ml [22] (Table 1).

**Table 1:** Perioperative outcomes of minimally invasive surgery in horseshoe kidney with ureteropelvic junction obstruction

	Bove [35] (N = 5)	Faddegon [34] (N = 9)	PingWang [22] (N = 8)	Jarzemski [36] (N = 4)	Shadpour [16] (N = 15)	Lu [21] (N = 5)
Age	37.4	18	23.9	14–47	17.7	32.8
Male	4/5	8/9	7/8	NS	11/15	4/5
Left side	2/5	8/9	5/8	3/4	12/15	NS
Primary UPJO	4/5	8/9	NS	3/4	NS	NS
Crossing vessel	2/5	7/9	NS	NS	8/15	NS
Technique	TPL	TP (TPL/RAL)	RPL	TPL	TPL	RPL
Isthmusectomy	None	None	Yes	Yes	Yes	Yes
Operative time (mean)	196	136	192	300	129	NS
Estimated blood loss (ml)	122	12.5	30	135	NS	NS
Length of stay (day)	3.2	1.5	5.8	9.25	2.8	5.7
Perioperative complication	None	None	None	None	None	None
Clinical success	100%	100%	100%	NS	93.3%	100%
Radiographic success	100%	78%	100%	NS	93.3%	100%
Recurrence	None	NS	None	NS	NS	NS

NS: Not Specified; TPL: Transperitoneal Laparoscopic; RPL: Retroperitoneal Laparoscopic; RAL: Robotic-Assited Laparoscopic

In this case, intraoperative dissection proved particularly challenging due to the presence of dense perinephric adhesions and the inherent technical complexity of the procedure. These factors, combined with limited early experience, contributed to a prolonged operative time of 270 min. Despite the extended duration, the procedure was completed successfully, with minimal blood loss and no major perioperative complications, thereby demonstrating the safety and feasibility of combining laparoscopic pyeloplasty with isthmusectomy.

Our operative time is consistent with the findings of Jarzemski et al. (2014), who reported durations ranging from 240 to 320 min in a series of four patients undergoing laparoscopic pyeloplasty with isthmusectomy for horseshoe kidney [36]. In contrast, shorter operative times have been reported in studies, where isthmusectomy was not performed. Bove et al. (2004) documented a mean operative time of 196 min in five cases of laparoscopic pyeloplasty without isthmusectomy, while Fedrigon et al. (2013) reported an even shorter mean duration of 136 min in nine similar cases [34, 35].

These comparisons underscore the fact that isthmusectomy, although not routinely required, adds significant technical complexity and contributes to prolonged operative time. Nevertheless, in appropriately selected cases—particularly those in which the isthmus contributes to ureteral obstruction or surgical inaccessibility—this step may be necessary to achieve optimal anatomical and functional outcomes. With increasing surgical expertise and careful patient selection, laparoscopic pyeloplasty combined with isthmusectomy can be safely and effectively performed in experienced surgeons.

Horseshoe kidney presents unique anatomical challenges in both the diagnosis and treatment of ureteropelvic junction obstruction (UPJO). A variety of diagnostic tools are required to accurately assess both structure and function, while treatment must be tailored to individual anatomy and underlying pathophysiology. Table 2 summarizes the current diagnostic modalities and treatment options available for managing UPJO in horseshoe kidneys. [3, 5, 9, 10, 14, 21].

**Table 2:** Summary of diagnostic modalities and treatment options for UPJO in horseshoe kidney

Category	Modality/method	Description
Clinical evaluation	History and Physical Exam	Congenital anomalies, flank pain, recurrent UTIs, hematuria, palpable mass (in rare cases)
	Symptom-based assessment	Often asymptomatic; symptoms typically due to complications (e.g., hydronephrosis, stones)
Imaging modalities	Ultrasound	Initial screening; detects hydronephrosis and renal morphology
	Intravenous Urography (IVU)	Outlines anatomy and delayed drainage; limited use today
	CT Urography	Provides detailed anatomy, identifies UPJO, stones, and aberrant vessels
	Magnetic Resonance Urography (MRU)	Non-radiation alternative; good soft tissue contrast
	Diuretic Renal Scintigraphy (e.g., MAG3, DTPA)	Functional assessment; confirms obstruction and differential renal function
Endoscopic assessment	Retrograde Pyelography	Visualizes ureter and UPJ; useful in complex anatomy or prior surgery
Treatment options	Conservative Management	For asymptomatic patients or non-obstructive dilation
	Open Anderson–Hynes Pyeloplasty	Gold standard; allows isthmusectomy, nephropexy when indicated
	Laparoscopic Pyeloplasty	Transperitoneal preferred; good visualization in anteriorly placed UPJ
	Robotic-Assisted Pyeloplasty	Enhanced precision; ideal for complex anatomy; limited availability
	Vascular Hitch (Hellström Technique)	For cases with crossing vessels without intrinsic obstruction
	Endopyelotomy (antegrade or retrograde)	Minimally invasive; limited success in complex or high-insertion cases
	Isthmusectomy	Selective use; indicated when isthmus causes mechanical obstruction

## Conclusion

Laparoscopic pyeloplasty with isthmusectomy is a feasible and minimally invasive approach for the management of UPJO in horseshoe kidneys, especially in cases involving vascular compression or isthmus-related obstruction. While the necessity of isthmusectomy remains a matter of debate, it may improve outcomes in selected patients. The use of an endoscopic stapling device facilitates safe and efficient division of the isthmus by providing simultaneous tissue transection and hemostasis. However, surgeon experience and careful patient selection remain critical to optimizing results and minimizing complications.

## Data Availability

The data that support the findings of this study are available from the corresponding author upon reasonable request.
